# Rethinking the Role of Radiation Therapy in the Management of Epithelial Ovarian Cancer

**DOI:** 10.3390/diagnostics10040211

**Published:** 2020-04-11

**Authors:** Geraldine Jacobson, Valerie Galvan-Turner

**Affiliations:** 1 Department of Radiation Oncology, West Virginia University, Morgantown, WV 26505-9234, USA; 2 Department of Obstetrics and Gynecology, West Virginia University, Morgantown, WV 26505-9234, USA; valerie.galvanturner@hsc.wvu.edu

**Keywords:** ovarian cancer, radiation therapy, stereotactic body radiation (SBRT)

## Abstract

Radiation has been relegated to a palliative role in the management of epithelial ovarian cancer (EOC). Contemporary radiation techniques, including intensity modulated radiation therapy (IMRT), stereotactic body radiation therapy (SBRT), and image-guided radiation therapy, enable conformal treatment that controls local disease with minimal morbidity. Recent studies from multiple institutions support the role of radiation in the ablative treatment of oligometastatic disease and control of locally recurrent and metastatic disease. Effective local treatment with radiation complements the role of systemic therapy in the management of EOC; reduces symptoms and disease burden, and may contribute to a prolonged drug free interval.

Physicians who care for patients with epithelial ovarian cancer (EOC) should reconsider the role of radiation in the management of this disease since contemporary radiation techniques, including intensity-modulated radiation therapy (IMRT), stereotactic body radiation therapy (SBRT), and image-guided radiation therapy, enable definitive targeted conformal treatment delivery to localized disease with minimal effects on normal tissue.

Standard, initial management for advanced EOC is primary surgical cytoreduction followed by systemic chemotherapy. The majority of patients will relapse after initial treatment and undergo further surgery and/or additional courses of systemic treatment.

Historically, radiation had been used to treat the entire peritoneal region at risk [1]. This approach was limited by the inability to deliver a curative radiation dose to an entire anatomic region, with multiple dose-limiting normal tissues. With the advent of effective chemotherapy, radiation therapy was relegated to a palliative role. However, the limited ability of radiation to control potential disease in the entire peritoneal cavity was not a sign of the ineffectiveness of radiation for treatment of EOC but rather a mismatch between the treatment modality and clinical goals. This perspective posits that radiation remains an effective modality for local control and should be considered complementary to systemic treatment. 

Involved field radiation therapy (IFRT) is a general term applied to a limited radiation field treating a defined tumor mass. This is in contrast to large field radiation, such as whole abdominal radiation, which was designed to encompass potential microscopic disease, historically used in the initial management of EOC. (1). 3-D conformal radiation therapy and intensity-modulated radiation therapy (IMRT) are planning methods that shape the radiation to the tumor and limit the dose to non-involved normal tissue. Stereotactic body radiation therapy (SBRT) delivers high doses in a short course (three–five treatments) using precise positioning and treatment delivery. The selection of a radiation modality for recurrent localized EOC is based on the location and size of the recurrence. 

A growing body of literature, discussed in this article, supports the efficacy of definitive radiation therapy for controlling local disease in patients with EOC, contributing to progression-free survival (PFS) and allowing prolonged periods of freedom from systemic treatment.

A retrospective study from the University of Texas MD Anderson Cancer Center of involved field radiation therapy (IFRT) for the treatment of patients with locally recurrent EOC reported a 5-year in-field control rate of 71% [2]. Patients were treated between 1999 and 2009 with a variety of planning methods, including IMRT and 3-dimensional (3D) conformal radiation therapy, with a median dose of 48 grays (Gy). Thirty-five of 102 patients had no evidence of disease (NED) at a median follow-up of 38 months; 25 patients were continuously disease-free for a median follow-up of 61 months [2].

A smaller, retrospective study by Albuquerque et al. (2016) of heavily pretreated patients with EOC receiving involved field radiation therapy (IFRT), from 40–60 Gy at 1.8–2 Gy per fraction (median dose 50.4 Gy) noted a local recurrence-free survival of 70% at 5 years and 60% at 10 years [3].

A review of IMRT in patients with locally recurrent EOC refractory to chemotherapy from Washington University treated with a median dose of 50.4 Gy, 1.8 Gy per fraction, reported a 2-year local control rate of 82% and overall survival of 63%, with limited radiation toxicity [4].

A multi-institutional, prospective trial from Korea evaluated the efficacy of IFRT in patients with locoregionally confined recurrent or persistent EOC. [5] All patients had 3D-based planning with 3-D conformal radiation, IMRT, or brachytherapy. The dose range was 45–66 Gy (mean dose 54 Gy) at 1.8–3 Gy per fraction. The complete response rate was 50%, and the overall response rate 85.7%. With a median follow-up of 28 months, median PFS (progression-free survival) was 7 months. In this study, patients with platinum-resistant malignancy did not have an inferior outcome compared with patients with platinum-sensitive disease [5].

Weichselbaum and Hellman (1995) proposed the concept of oligometastases, an intermediate state between localized disease and widespread metastases [6]. This concept applies to a limited number of discrete metastases, often defined as 1–3. The clinical implication of this concept is that aggressive local treatment of limited metastatic disease may contribute to progression-free survival (PFS). This concept is clinically supported by the positive impact on long term survival of resecting liver metastases from colon cancer [7] and pulmonary metastases from soft tissue sarcoma [8]. A review of 1001 consecutive cases of hepatic resection for metastatic colorectal cancer reported a 5-year survival of 37% and 10-year survival of 22%. Patients with a limited size and number of resected lesions had a more favorable outcome. [7]. A single-institution review of sarcoma patients who had complete surgical resection of pulmonary metastases demonstrated a 5-year survival of more than 50% [8].

SBRT is a precise, image-guided, ablative radiation modality that delivers a high dose of radiation to a limited area. SBRT has been used to control oligometastatic disease in multiple tumor sites with acceptable toxicity and improved survival [9,10,11]. Recent studies have explored the potential role of SBRT in gynecologic malignancies. 

A phase II clinical trial of SBRT (24 Gy in 3 fractions) in patients with recurrent gynecologic malignancies (50% ovarian cancer) showed a target response rate of 96% and median disease-free survival of 7.8 months [12].

Lazzari et al. (2018) reviewed a single-institutional experience of SBRT for recurrent oligometastatic disease in patients with ovarian cancer; the median dose was 24 Gy in 3 fractions. A complete radiologic response was noted in 60% of patients. In this study, the radiologic response was evaluated by the same imaging modality used for treatment planning, CT or PET-CT scan, and classified according to Response Evaluation Criteria in Solid Tumors (RECIST) version 1.1 or PET/CT Response Criteria in Solid tumors (PERCIST). The median systemic treatment-free interval after SBRT was 7.4 months, and the 2-year PFS rate was 68%. No patient was lost to follow up. In 69.5% of patients, no acute or late toxicities were observed. 23% of patients experienced acute grade 1 or 2 GI (gastrointestinal) toxicity, 28% of patients experienced grade 1 or 2 GI toxicity, or grade 1 genitourinary toxicity [13].

A large, multicenter Italian study (MITO RT-01) of SBRT in more than 400 patients with metastatic persistent and recurrent ovarian cancer reported a complete response in 65.2% of patients and a partial response in 23.8% of patients [14]. Mild toxicity was experienced in 20.7% of patients, of which 76% were grade 1 and 24% grade 2. This was a retrospective study designed to evaluate the activity and safety of this treatment. While it has the limitations of a retrospective single-arm study, it demonstrated a significant response rate and safety profile in this group of patients. 

The development of more effective systemic therapies, including cytotoxic, targeted, and immunotherapy, can potentially lead to significant cytoreduction and downstaging of patients with widely metastatic disease to oligometastatic disease, offering the potential for aggressive local treatment. Conversely, early-stage patients with prolonged survival may develop oligometastatic disease amenable to definitive local treatment. Both groups of patients may benefit from definitive SBRT. 

IMRT and conformal radiation therapy provide durable local control for persistent or recurrent local disease, even in patients who have failed several lines of chemotherapy. SBRT, a highly precise ablative treatment delivered in three to five treatments, can provide local control in oligometastatic disease. These radiation applications can reduce disease burden, relieve symptoms, and contribute to PFS and a prolonged drug holiday in EOC.

In summary, EOC is a disease of the entire peritoneal cavity, and as such, is best managed with an initial systemic approach. However, most patients will eventually relapse and require additional treatment. For relapses limited to a localized area, radiation therapy provides an effective local treatment modality that potentially complements the primary role of systemic therapy. For patients with limited sites of recurrence, radiation offers a potential break from systemic therapy while controlling symptoms and providing effective treatment.
